# The associations between daily spring pollen counts, over-the-counter allergy medication sales, and asthma syndrome emergency department visits in New York City, 2002-2012

**DOI:** 10.1186/s12940-015-0057-0

**Published:** 2015-08-27

**Authors:** Kazuhiko Ito, Kate R. Weinberger, Guy S. Robinson, Perry E. Sheffield, Ramona Lall, Robert Mathes, Zev Ross, Patrick L. Kinney, Thomas D. Matte

**Affiliations:** New York City Department of Health and Mental Hygiene, Bureau of Environmental Surveillance and Policy, New York, NY 10013 USA; Department of Environmental Health Sciences, Mailman School of Public Health, Columbia University, New York, NY 10032-3727 USA; Louis Calder Center, Biological Field Station, Fordham University, Armonk, New York, NY 10504-1104 USA; Department of Natural Sciences, Fordham College at Lincoln Center, 113 West 60th Street, New York, NY 10023 USA; Departments of Pediatrics and Preventive Medicine, Mount Sinai School of Medicine, 1 Gustave L. Levy Pl., Box 1512, New York, NY 10029 USA; New York City Department of Health and Mental Hygiene, Bureau of Communicable Disease, Queens, NY 11101 USA; ZevRoss Spatial Analysis, Ithaca, NY 14850 USA

**Keywords:** Spring tree pollen, Asthma emergency department visits, Allergy medication

## Abstract

**Background:**

Many types of tree pollen trigger seasonal allergic illness, but their population-level impacts on allergy and asthma morbidity are not well established, likely due to the paucity of long records of daily pollen data that allow analysis of multi-day effects. Our objective in this study was therefore to determine the impacts of individual spring tree pollen types on over-the-counter allergy medication sales and asthma emergency department (ED) visits.

**Methods:**

Nine clinically-relevant spring tree pollen genera (elm, poplar, maple, birch, beech, ash, sycamore/London planetree, oak, and hickory) measured in Armonk, NY, were analyzed for their associations with over-the-counter allergy medication sales and daily asthma syndrome ED visits from patients’ chief complaints or diagnosis codes in New York City during March 1^st^ through June 10^th^, 2002-2012. Multi-day impacts of pollen on the outcomes (0-3 days and 0-7 days for the medication sales and ED visits, respectively) were estimated using a distributed lag Poisson time-series model adjusting for temporal trends, day-of-week, weather, and air pollution. For asthma syndrome ED visits, age groups were also analyzed. Year-to-year variation in the average peak dates and the 10^th^-to-90^th^ percentile duration between pollen and the outcomes were also examined with Spearman’s rank correlation.

**Results:**

Mid-spring pollen types (maple, birch, beech, ash, oak, and sycamore/London planetree) showed the strongest significant associations with both outcomes, with cumulative rate ratios up to 2.0 per 0-to-98^th^ percentile pollen increase (e.g., 1.9 [95 % CI: 1.7, 2.1] and 1.7 [95 % CI: 1.5, 1.9] for the medication sales and ED visits, respectively, for ash). Lagged associations were longer for asthma syndrome ED visits than for the medication sales. Associations were strongest in children (ages 5-17; e.g., a cumulative rate ratio of 2.6 [95 % CI: 2.1, 3.1] per 0-to-98^th^ percentile increase in ash). The average peak dates and durations of some of these mid-spring pollen types were also associated with those of the outcomes.

**Conclusions:**

Tree pollen peaking in mid-spring exhibit substantive impacts on allergy, and asthma exacerbations, particularly in children. Given the narrow time window of these pollen peak occurrences, public health and clinical approaches to anticipate and reduce allergy/asthma exacerbation should be developed.

**Electronic supplementary material:**

The online version of this article (doi:10.1186/s12940-015-0057-0) contains supplementary material, which is available to authorized users.

## Background

Tree pollens have been associated with higher rates of allergic sensitization [1–3], over-the-counter allergy medication sales [4], prescribed medication use for allergic rhinitis, rhinosinusitus, and conjunctivitis [5], asthma symptoms [6], asthma emergency department (ED) visits [7–11], and asthma hospitalizations [12, 13]. Allergic rhinitis, a risk factor for increased asthma severity, with a reported prevalence of up to 100 % in those with allergic asthma [14], affects an estimated 10 ~ 30 % of the world population [15]. The estimated direct costs for allergic rhinitis alone, including both health services and prescription medication sales, were $11.2 billion in the U.S. in 2005 [16]. In a large survey of pediatric patients and parents of patients with allergy, 54 % reported the use of an over-the-counter (OTC) allergy medication during the past 4 weeks [17]. Asthma affects 1 in 12 people in the U.S. [18]. Emergency department visits for asthma account for a major fraction (~$1 billion in the U.S. in 2006) of the direct cost of asthma [19]. Thus, estimating the impacts of spring tree pollen on these outcomes is relevant to public health policy.

The observational epidemiological studies mentioned above found associations with either individual tree pollen types or total tree pollen counts. Of the seven U.S. and Canadian studies that examined the associations between pollen and asthma emergency department visits or hospitalizations [7–13], only two studies, a study in Atlanta, GA [9], and a ten-city study in Canada [13] evaluated the impacts of individual tree pollen types. However, the dominant taxa and mix of tree pollens vary from city-to-city. The impact of climate change on the relevant characteristics (i.e., season length, starting date, etc.) of tree pollens are also likely different from pollen to pollen and across different climatic regions [20]. Thus, to estimate the impacts of tree pollens on asthma and allergy health outcomes and to develop a public health policy to mitigate their adverse effects in the future, epidemiological analysis will need to consider individual pollen types, each of which may be impacted differently by climate change.

Previously, we reported that the peak dates (an 1/0 indicator variable, based on measurements around the estimated peak periods) of three tree pollen genera, maple, oak, and birch (but not elm) were associated with daily OTC allergy medication sales during the years 2003-2008 [4]. Since then, we have developed an extensive database of daily concentrations of spring tree pollens for years 2002-2012 by reviewing archived pollen slides. We believe that this daily pollen database has an uniquely high level of completeness compared to other available data (e.g., National Allergy Bureau recommends that its monitoring stations report 3 out of every 7 days), as we have all days of the week except for days where the sampler malfunctioned. Thus, the objective of this study is to investigate the multi-day association between daily measurements of individual spring tree pollen genera and the OTC allergy medication sales as well as asthma ED visits syndrome data (“asthma syndrome ED visits”), with a focus on the relative importance of individual pollen types, the timing and magnitude of their impacts, and the consistency between these allergy and asthma indicators, which we monitor on a daily basis as part of the City’s syndromic surveillance system for situational awareness.

## Methods

### Pollen data

Airborne pollen was collected with a Burkard volumetric spore trap (Burkard manufacturing Co., Rickmansworth, UK) located on the rooftop of Fordham University’s Louis Calder Biological Station in Armonk, NY, about 30 miles north of Manhattan. This station provides the closest long-term pollen record for the New York City (NYC) region. Most slides have exposure periods that run from around 9 am to 9 am (a sample that starts at 9 am today represents the measurement for today), except all samples in 2002 and March 1-14, 2004 (2 pm to 2 pm; a sample that starts at 2 pm today represents the measurement for tomorrow) and all samples in 2011 and 2012 (midnight to midnight sample). Trained counters conducted microscopic analysis of pollen. Resulting daily pollen counts were converted into concentrations in particles per cubic meter of air for the eleven years from 2002 to 2012.

We identified nine tree pollen genera for analysis on the basis of clinical significance in the U.S. and observed sensitization patterns in the Northeastern U.S. and NYC [3, 21, 22] (species name in italic): *Acer* (maple), *Betula* (birch), *Quercus* (oak), *Ulmus* (elm), *Fraxinus* (ash), *Platanus* (sycamore/London planetree), *Fagus* (beech), *Carya* (hickory), and *Populus* (poplar). We use the common name (e.g., maple) from here on. We chose a data analysis period of March 1^st^ through June 10^th^, 2002-2012 to cover peak periods of these pollen types. Missing values (7 %) were imputed using the average of surrounding values. The majority of the missing data (46 % of the 7 %) occurred consecutively in the beginning of the sampling period (before March 15^th^) when most of the pollen genera showed zero or very low measured values afterwards.

### Health outcome data

OTC allergy medication sales data: Data on OTC pharmacy sales are reported electronically to the New York City Department of Health (NYCDOH) on a daily basis from over 200 stores from a major pharmacy chain, disproportionately in Manhattan (the most densely populated borough of NYC). The number of pharmacies reporting sales data fluctuated day to day, but during the study period, about 20 to 25 % of stores in Manhattan reported data to NYCDOH. The following brand-name and generic products were classified as allergy medications: Alavert, Benadryl, cetirizine, Claritin, loratidine, Sudafed, Tavist, and Zyrtec, as well as other medications described with the word “allergy”. The unit of this allergy indicator is the number of units sold per day. On the average, the percentages of units sold in five boroughs were: Manhattan (75 %); the Bronx (3 %); Brooklyn (10 %); Queens (10 %); and Staten Island (2 %). Despite the disproportionate sales across boroughs, the daily sales counts during the spring study period were highly correlated across boroughs, ranging from r = 0.83 (Manhattan vs. Staten Island) to r = 0.98 (Brooklyn vs. Queens), indicating high spatial uniformity of temporal variations in this ecologic allergy indicator within the city. The coverage of stores changed in late 2011, and therefore, for OTC allergy medication sales data, analysis was limited to the years 2002-2011.

Asthma syndrome ED visits data: During the study period, NYCDOH electronically received data from 52 hospitals (~95 % of annual ED visits in NYC). Data files contain date of visit, age, sex, residential zip code, and free-text chief complaint (the patient’s own description of his/her illness). The ED visits data are used to investigate aberrations in various illnesses, including asthma, diarrhea, and influenza-like illness [23]. The ED visits data are categorized into exclusive “syndromes” based on the patient’s chief complaint, using an algorithm that scans the chief complaint field for character strings assigned to a syndrome. For asthma ED syndrome, the script searched for the word “asthma”, “wheezing”, “COPD”, their common misspelled analogues and International Classification of Diseases 9^th^ edition codes associated with asthma (because some hospitals report diagnosis codes). We analyzed asthma syndrome ED visits for all ages and by age group: (1) 0-4; (2) 5-17; (3) 18-39; (4) 40-64; and (5) 65+. Based on the physician-diagnosed asthma ED visits data during the years 2005-2009 available from the New York State’s Statewide Planning and Research Cooperative System, we found that the temporal correlations between the daily asthma syndrome ED visits (the data we used in this analysis) and the physician-diagnosed asthma ED visits were high (Pearson correlation = 0.95). The correlations for specific age-groups were also high except the oldest age group: 0.91, 0.96, 0.91, 0.83, and 0.53 respectively, for the age groups noted above. Thus, the asthma syndrome ED visits data are a very good indicator of daily variation in physician-diagnosed asthma ED visits, except for the elderly group.

The main difference between the OTC allergy medication sales data and asthma ED visits syndrome data in terms of their seasonal pattern is that, while the spring peaks are the most dominant feature of the OTC allergy medication sales data, asthma ED visits syndrome data also exhibit other seasonal variations such as sharp rise in September, with the counts remaining high through the winter period. However, the sharp spring peaks in counts are the common feature in these two outcomes and they clearly correspond to the spring pollen period (see Additional file 1: Figure S1).

### Weather data

Hourly weather data from LaGuardia Airport were obtained from National Climatic Data Center (http://www7.ncdc.noaa.gov/CDO/cdopoemain.cmd?datasetabbv=DS3505&countryabbv=&georegionabbv=&resolution=40). From the hourly observations, we computed daily mean temperature and daily average relative humidity.

### Air pollution data

Since previous studies in NYC found associations between asthma outcomes and both ozone and fine particles (PM_2.5_) during warm months [24, 25], we considered these air pollutants as potential confounders. We obtained gravimetrically-measured PM_2.5_ (24-h) and ozone (hourly) data from the U.S. EPA’s Air Quality Subsystem. For ozone, daily 8-h maximum values were first computed from hourly data. We then computed daily citywide average values from available multiple monitors following the approach used by Schwartz [26], which accounts for differences in variance and mean between sites.

### Data analysis

**Fig. 1:**
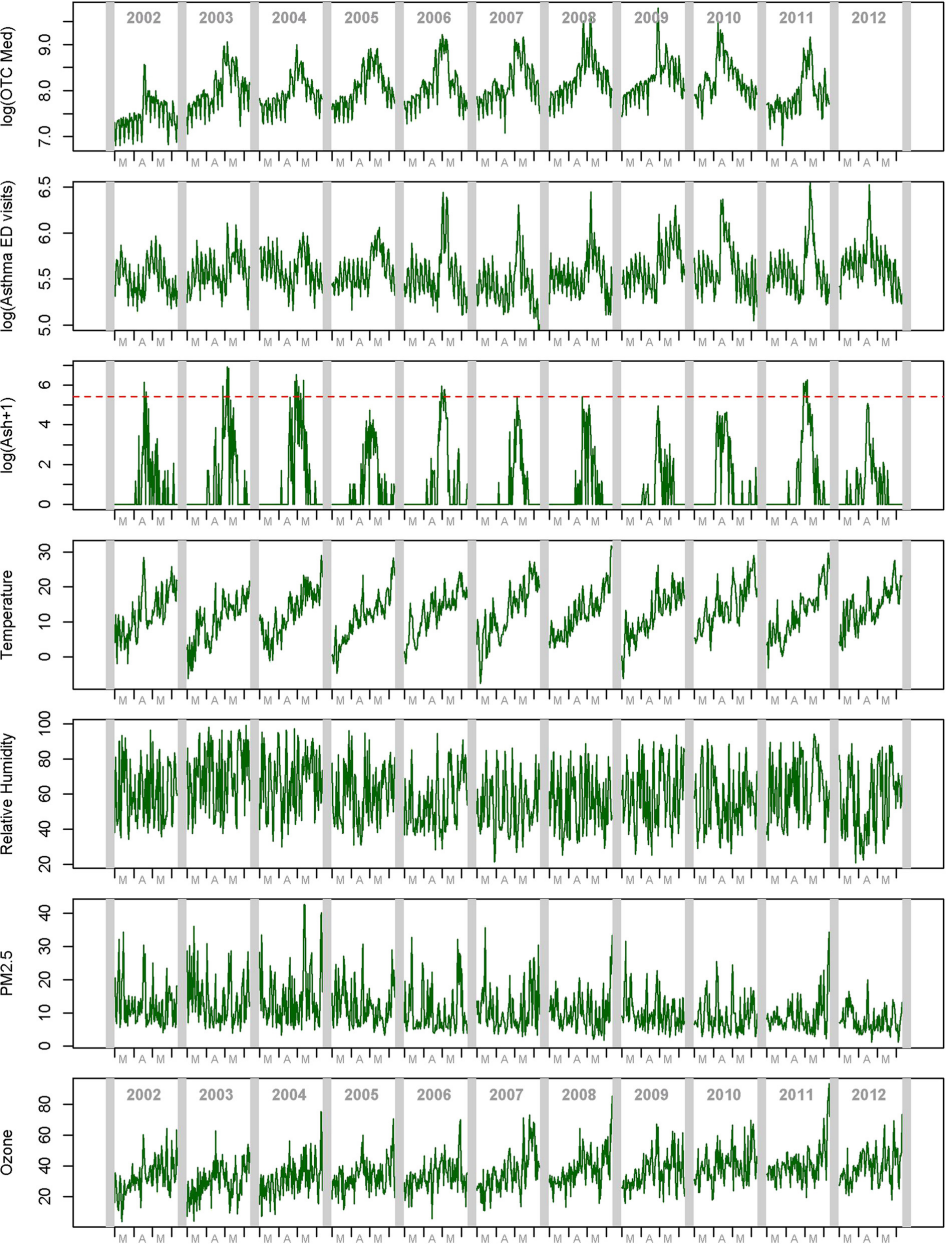
Time-series plots of OTC allergy medication sales, asthma syndrome ED visits, pollen (ash is shown), weather, and air pollution, during March 1 and June 10^th^, 2002-2012. The red horizontal dotted line for ash plot indicates 98^th^ percentile level

Figure 1 shows time-series plots of the outcomes, pollen (ash is shown), weather and air pollutants. Both tree pollen and air pollution concentrations are influenced by weather conditions such as temperature increase and rain, but unlike air pollution, each tree pollen type has a beginning and an end in each spring (i.e., flowering period), yielding an underlying broad bell-shaped concentration trend, which can be observed in Fig. 1. Therefore, in addition to time-series analysis, we also characterized the timing and duration of the tree pollen periods.

### Characterization of average peak calendar dates and year-to-year variation in pollen and the outcomes

First, to characterize the general timing of the pollen periods and the outcomes, we standardized each series so that its maximum height is 100 in each spring period. Then, each series was temporally smoothed with an 11-day moving average and then averaged across the study years by calendar date. The resulting values were plotted as density, yielding a “pollen calendar” with corresponding figures for the outcomes.

To assess if the year-to-year variation in seasonal peak date for pollen corresponds to those for the outcomes (i.e., does an early pollen season lead to an early peak in the allergy outcomes?), we computed the average calendar date of the five highest pollen levels in each season and the corresponding values for the outcomes, and computed Spearman’s rank correlation. However, asthma syndrome ED visits time-series exhibits two groups of major peaks within the March 1 – June 10^th^ study time window, with the first group occurring before mid-April and the second group after mid-April (see Fig. 1). The spikes in the first (earlier) group appear even before the levels of the early spring pollen type (elm and poplar) rise, suggesting that the first group of peaks in asthma syndrome ED visits is unlikely due to pollen. Therefore, to focus on the second group of peaks in asthma syndrome ED visits, which also generally coincide with the largest peaks of OTC allergy medication sales, for the outcomes, we considered the dates after April 15^th^ because the outcomes.

To assess if the duration of pollen season corresponds to those for the outcomes (i.e., does a shorter pollen season lead to a narrower peak in the allergy outcomes?), we defined the duration of the tree pollen season in each year as the days between the dates at 10^th^ and 90^th^ temporal cumulative percentiles of tree pollen concentrations and the corresponding values for the outcomes, and computed Spearman’s rank correlation. Again, for the outcomes, we considered the dates after April 15^th^ in computing the duration.

### Regression model to estimate the impacts of tree pollens on the allergy and asthma morbidity

Pollen exposure metrics: The distributions of all the tree pollen types were highly skewed. Our exploratory analysis of cross-correlation functions between pollen and the outcomes also showed that log-transformed pollen concentrations yielded better correlations than the raw data. Therefore, we log-transformed pollen concentrations (after adding 1) and used it as a pollen exposure metric. Since log-transformation was not commonly done in the past studies of pollen, we also evaluated sensitivity of results to using three alternative pollen metrics: (1) re-scaled in each spring by dividing the daily values by the maximum of that spring so that daily pollen intensity takes values between 0 and 100; (2) same as (1) but log-transformed; and, (3) raw data.

Regression model development: Based on preliminary evaluations of cross-correlation functions between the outcome and explanatory variables, we developed a regression model to estimate multi-day effects rate ratios, up to 3-day lags for OTC allergy medication sales and up to 7-day lags for asthma syndrome ED visits at 98^th^ percentile distribution of pollen levels compared to 0^th^ percentile. We express rate ratios per zero-to-near-the-peak (98^th^ percentile) increase in pollen levels because these two allergy indicators exhibit very sharp peaks every spring, distinctly sharp in comparison to other seasonal variations in the rest of the year (see Additional file 1: Figure S1), clearly in response to the peak levels of pollen. Of the eleven springs, the number of times the 98^th^ percentile level was achieved or exceeded were 8 for elm, 8 for poplar, 7 for maple, 7 for birch, 5 for beech, 6 for ash, 9 for sycamore/London planetree, 10 for oak, and 6 for hickory, indicating that this is a near-peak level that are observed in most of the springs for most of the pollen types we analyzed. We estimated cumulative rate ratios of the pollens’ impacts with a distributed lag model using the R package ‘dlnm’ [27] with a quasi-Poisson model accommodating over-dispersion, specifying an unconstrained lag form and a linear functional form. The model also adjusted for: (1) day-of-week; (2) within-season temporal trends using natural cubic splines of study days with 6° of freedom (d.f.) (determined by examining residual autocorrelation) per each spring; (3) daily average relative humidity with the same length of lags as the outcome, unconstrained for the lag form and natural cubic splines with 2 d.f. for the functional form; (4) daily average temperature fitted in the same way as relative humidity; (5) PM_2.5_ with up to 2-day lags with unconstrained lag form and a linear functional form; and (6) ozone with up to 2-day lags fitted in the same way as PM_2.5_. We applied the same model for the four age groups of asthma syndrome ED visits.

We conducted additional analyses to examine sensitivity of results to alternative model specifications for both outcomes: (1) using a third-degree polynomial distributed lag form rather than unconstrained lag form; (2) using a more aggressive adjustment for within-season temporal trends by using 8 d.f. per season; (3) without adjustment for temperature and relative humidity; and (4) without adjustment for PM_2.5_ and ozone.

All the analyses were conducted using R statistical package version 3.03 (R Core Team, Vienna, Austria, 2014). The significance of statistical tests in the following text is based on alpha = 0.05 and two-tailed test.

The Institutional Review Board of NYCDOH approved the study protocol.

## Results

Data distributions of daily values for all the variables are shown in Table 1. All the tree pollen types are highly skewed in part because the tree pollen concentrations occur in a limited time window within the March 1^st^ – June 10^th^ period. Correlation among the pollen types ranged from weakly negative to moderately positive (shown in the Additional file 1: Table S1).

**Table 1:** Data distribution during March 1^st^ through June 10^th^, 2002-2012 (2002-2011 for OTC allergy medication sales)

Variables	Mean	S.D.	Min.	25 %	50 %	75 %	98 %	Max.
OTC Allergy Medication Sales (# units sold/day)	3493	1953	901	2263	2943	4086	9565	17971
Asthma Syndrome ED Visits (visits/day)								
All Ages	269.0	71.6	104	222	254	298	468	704
Age 0-4	47.5	11.7	15	39	47	55	73	100
Age 5-17	64.2	30.8	18	43	58	77	155	243
Age 18-39	65.1	23.0	27	51	60	73	133	215
Age 40-64	72.0	18.1	32	60	70	81	122	169
Age 65+	20.1	5.7	7	16	20	24	32	47
Spring Tree Pollen (grains/m^3^)								
Elm	8.7	39.9	0	0	0	2.2	97.2	624.7
Poplar	6.7	25.8	0	0	0	1.8	77.3	380.1
Maple	15.3	101	0	0	0	7.2	130.0	3091.2
Birch	142.8	649.1	0	0	1.8	21.6	1680.3	10235.3
Beech	2.6	11.6	0	0	0	0	30.9	162.0
Ash	17.9	73.1	0	0	0	2.2	223.3	1029.7
Syc./London plane	9.4	43.6	0	0	0	0	97.2	696.0
Oak	190.6	683.9	0	0	2.1	57.0	1932.1	13040.4
Hickory	11.3	41.4	0	0	0	2.2	131.4	768.9
Weather								
Temperature (°C)	12.5	6.7	-7.7	8.1	12.6	17	26.3	31.8
Rel. Humid. (%)	59.7	17.4	20.8	44.6	58.8	73.8	93.3	99.3
Air Pollutant								
PM_2.5_ (μg/m^3^)	10.9	6.4	1.2	6.3	9.1	13.8	28.6	42.6
Ozone (ppb)	35.7	11.8	3.9	28.4	34.8	41.8	66.7	93.5

**Fig. 2:**
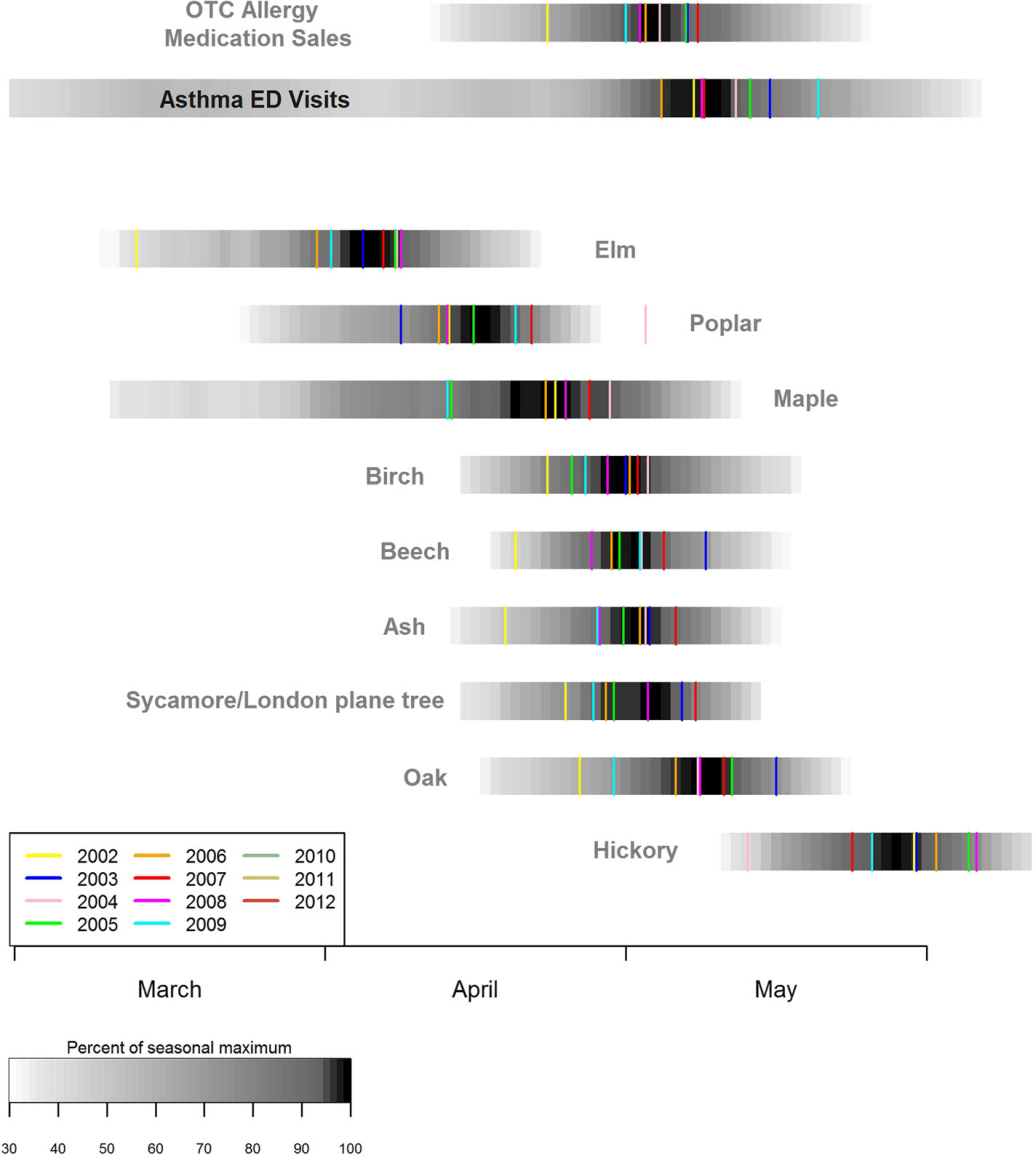
Average intensity of OTC allergy medication sales, asthma syndrome ED visits, spring tree pollens smoothed with an eleven-day moving average (and log-transformed for tree pollens) averaged by calendar date across eleven years (2002-2012; 2002-2011 for the medical sales data). Average calendar dates of individual years’ five highest levels are also shown

Figure 2 shows the intensity of the level of tree pollens averaged across eleven years. The average peak calendar dates of individual years are also superimposed. The estimated average peak dates and durations are shown in Table 2. The average peak date (May 2^rd^) for OTC allergy medication sales occurs a few days after the average peak dates of mid-spring pollens, and the average peak dates for asthma syndrome ED visits (May 11^th^) follows that for OTC allergy medication sales. Maple showed the longest average duration (25 days), while the mid-spring tree pollens showed shorter durations. Thus, the year-to-year variability in average peak calendar dates, especially for the mid-spring pollens, is small (i.e., standard deviation < 10 days).

**Table 2:** Characteristics of tree pollen peaks and their correlation with the outcomes

Tree Pollen Type/outcome	Average peak calendar dates^a, b^ (S.D.)		Average 10^th^-to-90^th^ % Duration^b, c^ days (S.D.)		Rank Correlation^d^ of Peak dates		Rank Correlation^d^ of Duration	
					OTC Allergy Med.	Asthma syndrome ED visits	OTC Allergy Med.	Asthma syndrome ED visits
Elm	31-Mar	(10.6)	15.0	(10.1)	0.55	0.50	-0.37	-0.38
Poplar	14-Apr	(9.9)	17.0	(6.9)	0.35	0.60	-0.15	-0.20
Maple	18-Apr	(13.5)	25.1	(10.2)	0.62	0.37	-0.28	-0.29
Birch	27-Apr	(5.7)	17.1	(9.6)	**0.67**	0.40	**0.76**	**0.88**
Beech	30-Apr	(5.7)	18.9	(6.1)	**0.80**	**0.74**	**0.73**	**0.84**
Ash	28-Apr	(7.3)	12.6	(4.0)	**0.90**	0.49	0.19	0.33
Sycamore/London planetree	28-Apr	(8.8)	12.5	(4.1)	**0.91**	0.51	0.32	0.49
Oak	4-May	(9.3)	12.5	(5.5)	**0.92**	**0.66**	**0.86**	**0.85**
Hickory	27-May	(6.7)	17.8	(5.7)	0.09	0.05	0.49	0.42
OTC Med Sales	2-May	(7.9)	44.7	(2.2)	NA	0.47	NA	**0.89**
Asthma Syndrome ED visits	11-May	(9.9)	46.5	(1.4)	0.47	NA	**0.89**	NA

Note: ^a^For each spring, the average peak date is defined as the average calendar date of the five highest pollen concentrations; ^b^For the outcomes, this computation was done for on and after April 15^th^; ^c^For each spring, the 10^th^-to-90^th^ percentile duration is the period between the dates with the 10^th^ and 90^th^ cumulative sum percentile of concentrations; ^d^Spearman’s rank correlation between the average peak date or duration of tree pollen vs. that of the outcome across years. The bold-faced correlations are significant at alpha = 0.05 level

Among Spearman rank correlations of the correspondence between the peak dates of pollens and those for OTC allergy medication sales, birch, beech, ash, sycamore/London planetree, and oak were statistically significant; and beech and oak were significant for asthma syndrome ED visits (Table 2). Diagrams of temporal correspondence between peaks of tree pollens and OTC allergy medication sales in each of the ten years are shown in the (Additional file 1: Figure S2). Spearman rank correlations between the estimated durations also showed significant relationships for birch, beech, and oak for both outcomes, and the durations of the two outcomes were also significantly correlated.

**Fig. 3:**
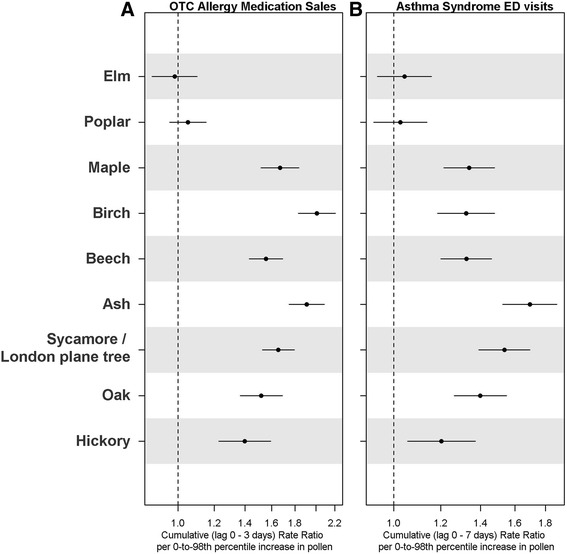
Cumulative rate ratios per 0-to-98^th^ percentile increase in spring tree pollen levels for: (**a**) OTC allergy medication sales (0-3 days); and (**b**) asthma syndrome ED visits (0-7 days)

Figure 3 shows the estimated cumulative rate ratio for both outcomes per 0-to-98^th^ percentile increase for each of the nine pollen types. Neither elm nor poplar was significantly associated with these two outcomes. The magnitude of associations is substantial, especially mid-spring tree pollen types (maple, birch, beech, ash, oak, and sycamore/London planetree) with cumulative rate ratios up to 2.0 for OTC allergy medication sales (e.g., 1.9 [95 % CI: 1.7, 2.1] for ash pollen) and up to 1.7 for asthma syndrome ED visits (e.g., 1.7 [95 % CI: 1.5, 1.9] for ash pollen). The lagged associations for asthma syndrome ED visits were longer than those for OTC allergy medication sales (shown in the Additional file 1: Figure S3).

**Fig. 4:**
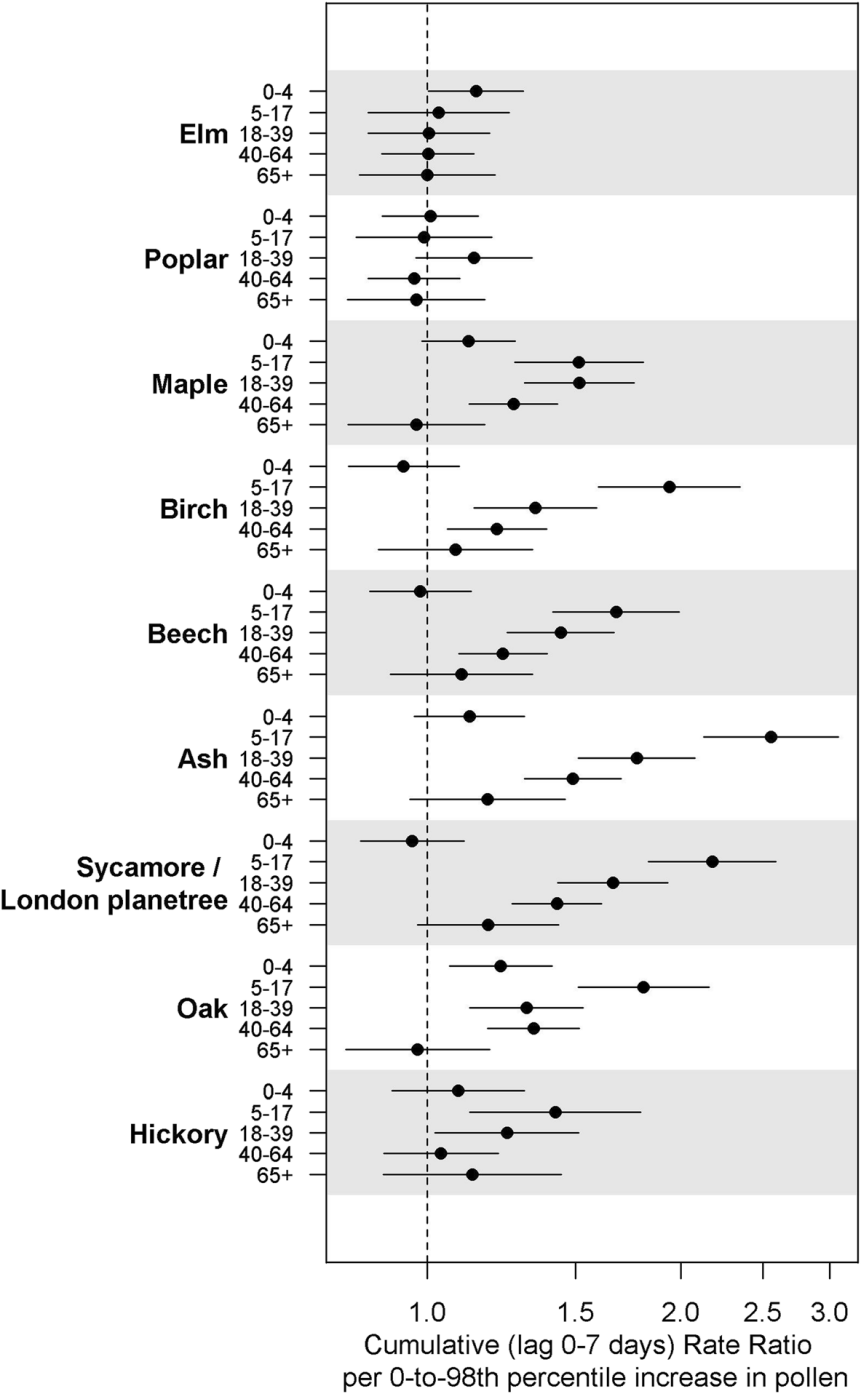
Cumulative rate ratios per 0-to-98^th^ percentile increase in spring tree pollen levels for asthma syndrome ED visits (0-7 days) by age groups

The analysis of asthma syndrome ED visits by age group found strong age-dependency (Fig. 4). For most of the tree pollens, the 5-17 age group showed the strongest associations (e.g., a cumulative rate ratio of 2.6 [95 % CI: 2.1, 3.1] per 0-to-98^th^ percentile increase for ash pollen), while the associations were generally null for both the 0-4 and 65+ age groups.

Of the two air pollutants, ozone was consistently and statistically significantly associated with both OTC allergy medication sales and asthma syndrome ED visits (results not shown). For example, the largest estimated cumulative rate ratio for ozone at lag 0 through 2 days for OTC allergy medication sales was 1.12 (95 % CI: 1.08, 1.16) with oak in the model, per 10 ppb increase. In contrast, PM_2.5_ showed small but negative associations with OTC allergy medication sales (which were sensitive to weather adjustment, as the associations were positive without the weather terms) and consistently null associations with asthma syndrome ED visits during the spring season (results not shown).

The alternative pollen metrics generally showed comparable rate ratios for both outcomes except for the raw data, which showed the poorest associations with both outcomes (result shown in the Additional file 1: Figure S4).

The estimated tree pollen rate ratios for both OTC allergy medication sales and asthma syndrome ED visits were generally insensitive to alternative model specifications in terms of a lag form of pollen, a more aggressive temporal adjustment, and adjustment for weather and air pollution (shown in the Additional file 1: Figure S5).

## Discussion

Our study analyzed more individual clinically-relevant spring tree pollen types than any of the past observational time-series studies of allergy/asthma outcomes. Our study is also unique in that two allergy/asthma outcomes were analyzed using a consistent approach. We found that mid-spring pollen types (maple, birch, beech, ash, sycamore/London plane tree, and oak) showed the strongest associations with both OTC allergy medication sales and asthma syndrome ED visits consistently. Associations were even strongest in children (ages 5-17). These findings add to the existing evidence from the population-based studies of the impacts of tree pollens on allergy/asthma outcomes, but some aspects of our analyses are more elaborate and merit further discussion.

### Timing, duration, and magnitude of association

Our rank correlation analysis of the year-to-year correspondence between the peak calendar dates for pollen and the outcomes indicates that the timing of the peak abundance period for some of the mid-spring pollen types were significantly associated with the corresponding timing for OTC allergy medication sales (birch, beech, ash, and sycamore) and asthma ED visits (beech and oak), implying that when the pollen peak appeared early in the spring, so did the peaks of the outcomes. Likewise, the rank correlation results of the year-to-year correspondence in the 10^th^-to-90^th^ temporal cumulative durations indicate that the durations of birch, beech, and oak were significantly associated with those for both outcomes, implying a shorter flowering period of these pollen types would lead to a shorter associated period of elevated counts of these outcomes. While the relatively small sample size of these rank correlations of year-to-year variation (n = 10 for the medication sales data, *n* = 11 for the asthma syndrome ED visits data) limits the interpretation of these results, they provide an alternative and complementary approach to the usual time-series analysis of daily data, which we also conducted.

The estimated magnitude of the mid-spring tree pollens’ impacts on OTC allergy medication sales (cumulative rate ratio up to 2.0 per 0-to-98^th^ percentile increase) and asthma syndrome ED visits (up to 1.7 for all-age; up to 2.6 for age 5-17 group) is quite “large” for a temporary varying environmental risk factor and certainly larger than those for air pollution, though, unlike air pollution, the durations of these tree pollens are limited (e.g., 2 to 4 weeks) per year. We reviewed other recent studies that examined tree pollen impacts on allergy and asthma outcomes, and found that, while direct comparisons are difficult because rate ratios are computed for different increments (e.g., inter-quartile-range), these studies also reported substantive impacts (e.g., rate ratios > 1.2 had they been computed per 0-to-98^th^ percentile increase) [5, 9, 11]. Our rate ratios may also be larger compared to those reported in the past studies in part because they are cumulative rate ratios over multiple days with longer lags (i.e., 0-7 days for asthma syndrome ED visits) than previously considered in other studies.

### Coherence of associations with tree pollens between the two outcomes

We see coherence in the associations between tree pollens and OTC allergy medication sales and asthma syndrome ED visits in that they were both most strongly associated with mid-spring tree pollens and not associated with the early-spring pollens (elm and poplar). The average peak date for OTC allergy medication sales occurs about 9 days before the average peaks date for asthma syndrome ED visits, and the associations with tree pollens for asthma syndrome ED visits exhibit longer lags than for OTC allergy medication sales. The rank correlation analysis indicated that the elevated durations of these outcomes were significantly correlated, but not their average peak dates. These results may suggest that elevated levels of tree pollens induce and/or exacerbate allergic rhinitis in both asthmatic and non-asthmatic populations, but these two outcomes need not have happened in the same individuals. We currently do not have information on consumer reasons for the temporal increase in purchase of these medications (i.e., as a preventive measure or in response to symptoms onset). The OTC allergy medication sales averaged about 3,500 (representing only ~20 % coverage of retail drug stores in the city) sales per day, whereas asthma syndrome ED visits average about 260 visits per day (with ~95 % ED visits coverage in the city) during the spring period.

### Exposure misclassification

One major limitation of our study was potential exposure misclassification from using data from a monitor outside of New York City. A recent review [28] also suggests that within-city variation of tree pollen levels can be substantive. However, in our previous analysis of peak dates of three tree pollens (birch, maple, and oak) and OTC allergy medication sales in years 2003-2008, the estimated impacts were similar when the sales from each of five boroughs were separately analyzed, suggesting that the tree pollen data from outside the city can serve as a useful indicator of exposures to these tree pollens [4]. We speculate that the timing of pollen release is not significantly different between the trees in the city and those outside the city within the metro area, as shown in other locales [29]. Nevertheless, the nature and the extent of possible exposure errors for the tree pollens in our setting have not been characterized. The extent of such exposure misclassification error may also be different across these tree pollens. Therefore, some of us are currently investigating this issue by measuring pollen counts daily in the central NYC location and also collecting data using integrated pollen trap samplers at 45 locations within NYC (Weinberger et al., unpublished data) using the infrastructure of New York City Community Air Survey [30]. Our current analysis also expressed the rate ratio per near-peak (i.e., 98^th^ percentile) pollen levels rather than per grains/m^3^ to minimize a potential “scaling error” that can result from the influence of local trees on the pollen levels measured at a single location.

### Air pollution effects

The lack of PM_2.5_ association in this analysis may be due to the relatively low concentrations in the spring study period. In contrast, ozone was consistently associated with both outcomes, though the magnitude of its effect size was smaller than that for tree pollens. Past controlled studies involving asthmatic subjects suggest synergistic effects of ozone and allergens [31–34]. Ozone may also modify allergenicity of pollens [35, 36]. However, we were unable to specifically test an interaction effect of ozone and tree pollen because there were not sufficient days of high and low ozone with and without tree pollen peaks.

### Relative importance of tree pollen types

We found that the mid-spring tree pollens were most consistently and strongly associated with both OTC allergy medication sales and asthma syndrome ED visits. These two allergy outcomes were also consistently not associated with elm and poplar. These results are in part consistent with the results from the study of allergy patients in New York City using skin prick test [3], which concluded that the hypersensitivity to tree pollens most often was manifested with allergy to oak, birch, and maple, and that identifying beech, poplar, and elm hypersensitivity added little towards identifying patients who are allergic to tree pollen.

There is a limitation to linking the relative strength of observed associations across the tree pollens to their corresponding sensitization potencies. Because these tree pollens’ peaks appear in a generally expected order (e.g., elm the earliest and hickory the last), those whose peaks appear later in the spring (e.g., hickory) may not appear to have associations only because the largest impacts might have already taken place (i.e., the allergy medications purchased; subjects have visited ED so that the conditions have been controlled) from the pollens that appear earlier in the spring. Also, the mid-spring tree pollens may appear to have the largest impacts on these outcomes possibly because their concentrations are simultaneously high during the relatively narrow time window. Thus, the apparent relative impacts of tree pollens on allergy/asthma outcomes may depend on the dominant or mix of pollen taxa in a given city or region.

### Age dependence of association

We found that the tree pollens’ associations with asthma syndrome ED visits were strongest in children (age 5-17). The weaker associations for the very young (age 0-4) and the elderly (age 65+) may be due to greater uncertainty in identifying (as reported by self or family members) asthma in these age groups. Of the asthma ED visits studies that also examined multiple age groups, Darrow et al. [9] also found that age 5-17 group showed the strongest associations with oak compared to age groups 0-4 or 18 +.

### Climate change implications

Public health impacts of pollen could be affected in a number of different ways as climate changes in coming years. It has been hypothesized that climate change may contribute to the rise in asthma [37]. Specific global warming adaptation measures such as urban tree planting to combat urban heat island could also impact population level exposure to allergenic pollen. General guidelines for the choice of urban trees have been proposed such as the use of low pollen producing species [38], but specific choice of tree(s) for a given city may need to consider relative importance of pollen types that have impacts on asthma/allergy outcomes observed, such as we have presented here. Our result suggests that changes in timing of pollen season onset will influence timing of increase in allergy/asthma outcomes. However, assessment of the impact of climate change on tree season length and pollen abundance was outside the scope of this analysis.

## Conclusions

Tree pollens peaking in mid-spring exhibit substantive impacts on allergy and asthma morbidity. The pollen’s association with asthma syndrome ED visits was most pronounced in children. Given the relatively narrow time window of these pollens’ peak occurrences, public health and clinical approaches to anticipate and reduce allergy and asthma morbidity from spring pollen should be developed. Because the dominant taxa and mixture of tree pollens vary from region to region, similar assessment of the impacts of individual tree pollens on allergy and asthma outcomes would be beneficial in developing city/region specific public health programs to mitigate the adverse impacts of tree pollens and to plan for the future impact of climate change on tree pollens.

## Additional file

Additional file 1: Table S1 Correlation matrix of log-transformed tree pollens, weather, and air pollution variables. **Figure S1**: Year-round daily time-series of over-the-counter allergy medications sales, asthma ED visits syndrome, and log-transformed ash pollen concentrations. **Figure S2**: Year-to-year average peak date correspondence between tree pollens and OTC allergy medication sales. **Figure S3**: Individual lag days’ rate ratios for OTC allergy medication sales and asthma syndrome ED visits. **Figure S4**: Sensitivity analysis using alternative pollen metrics for OTC allergy medication sales and asthma syndrome ED visits. **Figure S5**. Sensitivity analysis using alternative model specifications for OTC allergy medication sales and asthma syndrome ED visits. (PDF 1467 kb)

## Abbreviations

ED: Emergency department
NYC: New York City
NYCDOH: New York City Department of Health and Mental Hygiene
OTC: Over-the-counter
PM_2.5_: Particulate Matter with aero-dynamic diameter equal to or less than 2.5 micrometer
CI: Confidence interval
