# Supplementary material for: The associations between daily spring pollen counts, over-the-counter allergy medication sales, and asthma syndrome emergency department visits in New York City, 2002-2012
Source: Environ Health. 2015 Aug 27;14:71. doi: 10.1186/s12940-015-0057-0 (PMC4549916; doi:10.1186/s12940-015-0057-0)
Supplement: Additional file 1: Table S1 — Correlation matrix of log-transformed tree pollens, weather, and air pollution variables. Figure S1: Year-round daily time-series of over-the-counter allergy medications sales, asthma ED visits syndrome, and log-transformed ash pollen concentrations. Figure S2: Year-to-year average peak date correspondence between tree pollens and OTC allergy medication sales. Figure S3: Individual lag days’ rate ratios for OTC allergy medication sales and asthma syndrome ED visits. Figure S4: Sensitivity analysis using alternative pollen metrics for OTC allergy medication sales and asthma syndrome ED visits. Figure S5. Sensitivity analysis using alternative model specifications for OTC allergy medication sales and asthma syndrome ED visits. (PDF 1467 kb) [file 12940_2015_57_MOESM1_ESM.pdf]

Additional supporting data for:

The associations between daily spring pollen counts, over-the-counter allergy medication sales, and asthma emergency department visits in New York City, 2002-2012.

Ito K, Weinberger KR, Robinson GS, Sheffield PE, Lall R, Mathes R, Ross Z, Kinney PL, and Matte TD.

Table of Contents (each item on a separate page):

(1) Table S1: Correlation matrix of log-transformed tree pollens, weather, and air pollution variables.
(2) Figure S1: Year-round daily time-series of over-the-counter allergy medications sales, asthma ED visits syndrome, and log-transformed ash pollen concentrations

(3) Figure S2: Year-to-year average peak date correspondence between tree pollens and OTC allergy medication sales.

(4) Figure S3: Individual lag days' rate ratios for OTC allergy medication sales and asthma syndrome ED visits.

(5) Figure S4: Sensitivity analysis using alternative pollen metrics for OTC allergy medication sales and asthma syndrome ED visits.

(6) Figure S5. Sensitivity analysis using alternative model specifications for OTC allergy medication sales and asthma syndrome ED visits

Table S1. Correlation matrix of daily exposure variables: log-transformed tree pollens, weather, and air pollutants during March 1 – June 10, 2002-2012. “Sycamore” is “Sycamore/London planetree”.

	Elm	Poplar	Maple	Birch	Beech	Ash	Sycamore	Oak	Hickory	Temperature	RH	PM2.5	O3
Elm	1												
Poplar	0.33	1											
Maple	0.31	0.48	1										
Birch	-0.15	0.13	0.43	1									
Beech	-0.15	0.04	0.34	0.76	1								
Ash	-0.15	0.12	0.44	0.82	0.71	1							
Sycamore	-0.1	0.09	0.31	0.70	0.64	0.69	1						
Oak	-0.24	-0.01	0.13	0.64	0.57	0.61	0.55	1					
Hickory	-0.29	-0.22	-0.22	-0.04	-0.03	-0.01	-0.07	0.17	1				
Temperature	-0.23	0.02	0.02	0.27	0.19	0.23	0.18	0.30	0.57	1			
RH	-0.09	-0.17	-0.22	-0.16	-0.12	-0.12	-0.13	-0.03	0.01	0.03	1		
PM2.5	0.04	0.02	-0.04	-0.03	-0.08	-0.04	-0.07	-0.04	0.13	0.26	0.23	1	
O3	-0.11	0.07	0.11	0.16	0.11	0.16	0.13	0.11	0.40	0.56	-0.35	0.06	1

Figure S1:

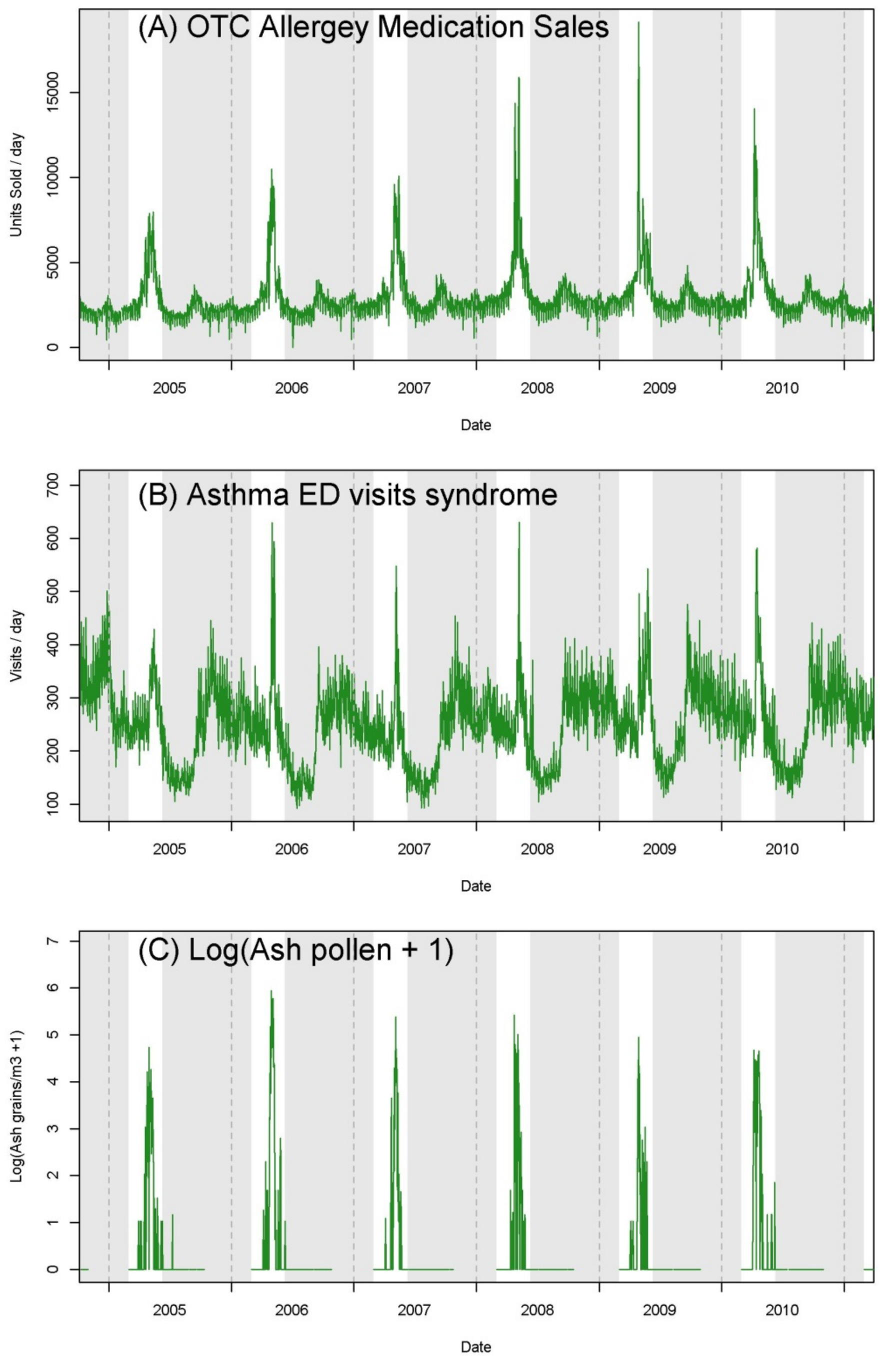

Year-round daily time-series plots of over-the-counter allergy medication sales, asthma ED visits syndrome, and log-transformed ash pollen levels, 2005-2010. The white areas are the spring study periods (March 1 – June 10).

Figure S2:

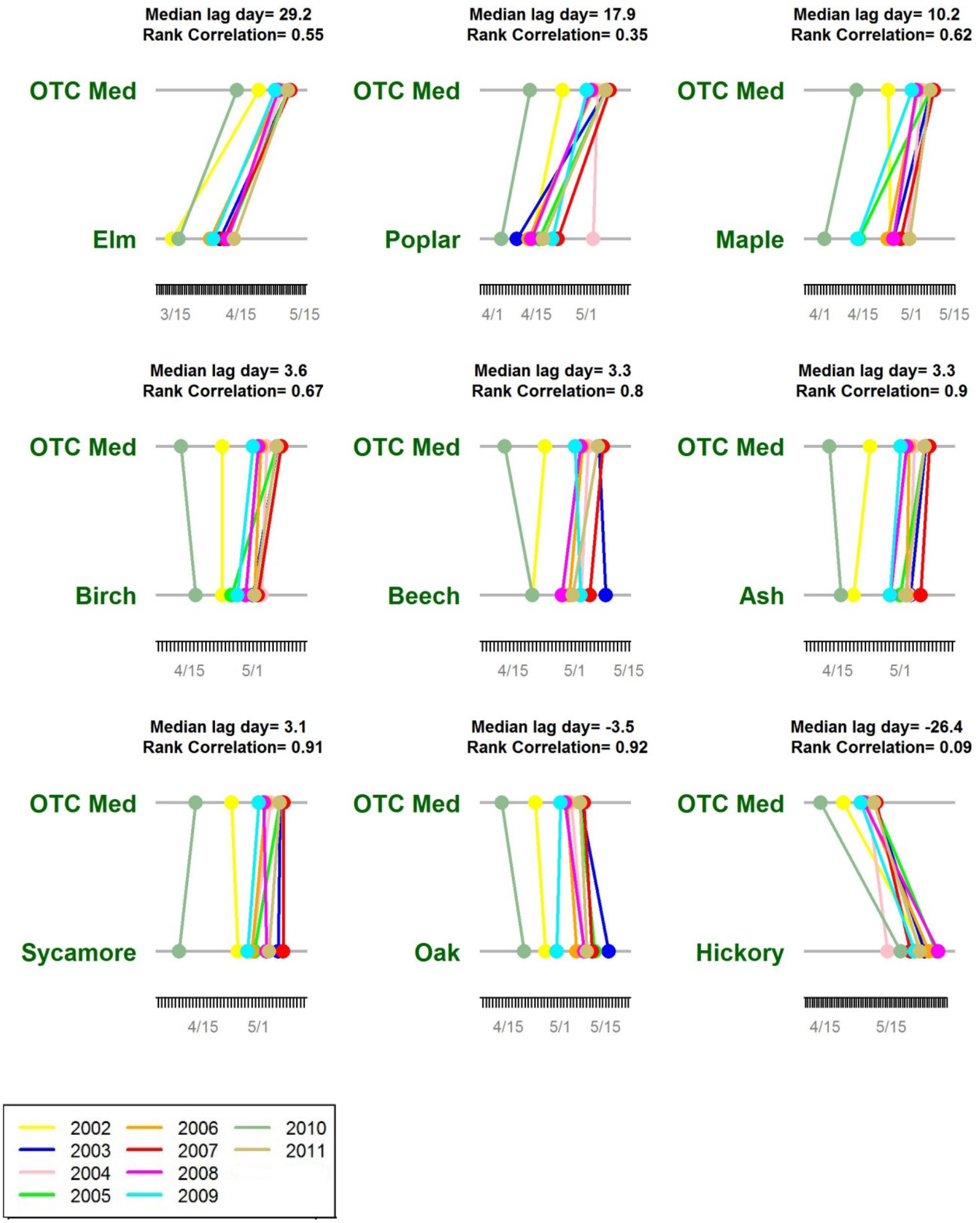

Year-to-year average peak calendar date correspondence between tree pollens and OTC allergy medication sales in years 2002 through 2011. The average peak calendar date is the average calendar date of five highest daily pollen concentrations (or largest OTC allergy medication sales). The label noted as “Sycamore” below is “Sycamore / London plane tree”.

Figure S3:

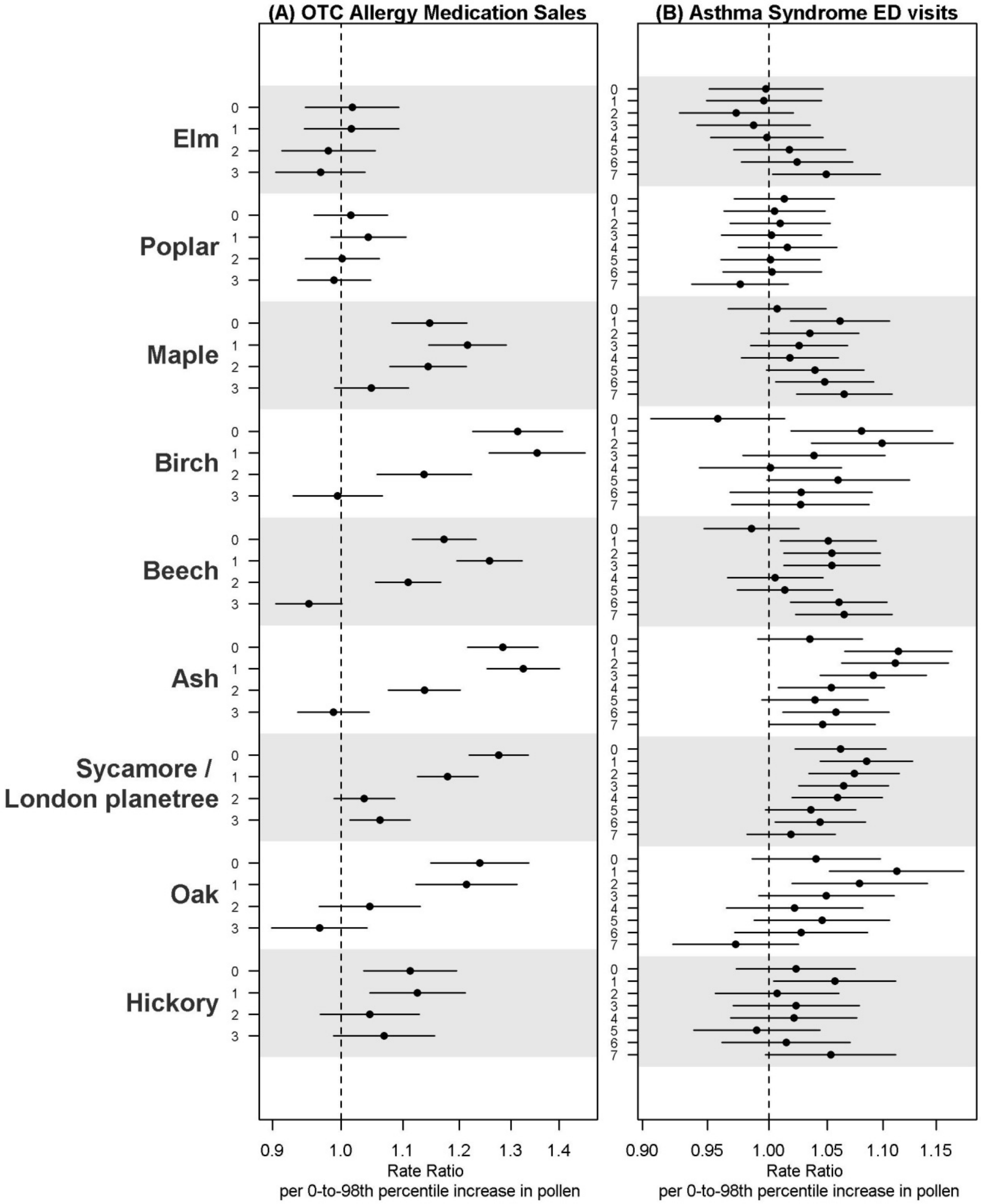

Rate ratios for individual lag days from distributed lag models per 0-to-98th percentile increase in pollen concentrations for (A) Over-the-counter (OTC) allergy medication sales (lag 0 through 3 days; years 2002-2011); and (B) asthma syndrome ED visits (lag 0 through 7 days; years 2002-2012).

Figure S4.

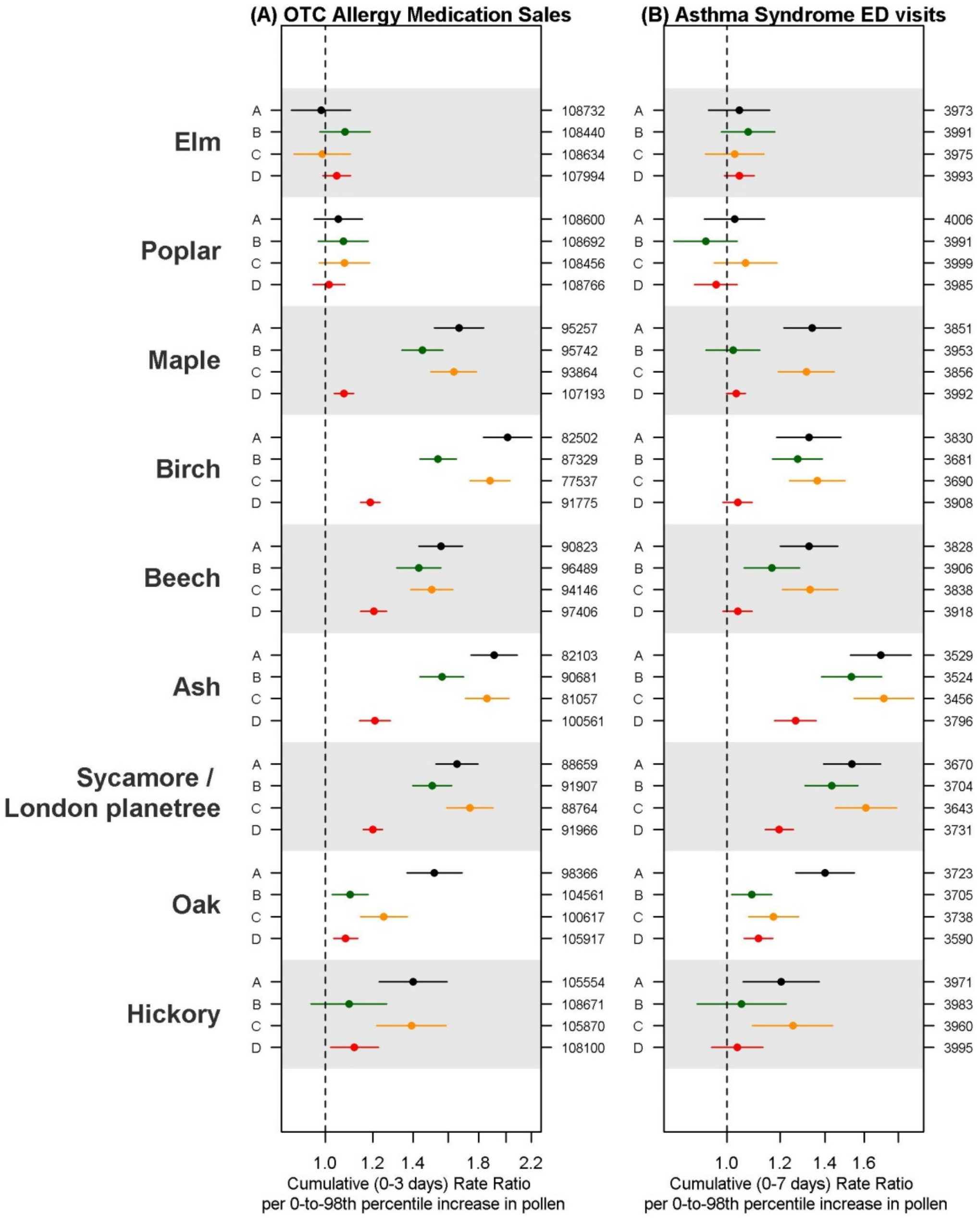

Sensitivity of estimated cumulative rate ratios to alternative pollen metrics for OTC allergy medication sales (left) and asthma syndrome ED visits (right). Pollen metrics: (A, main analysis): log-transformed; (B) standardized for year-to-year variation but not log-transformed; (C) standardized for year-to-year variation and log-transformed; and (D) raw data. The numbers on the right side of each figure are deviance of the model. Rate ratios were computed per 0-to-98th percentile increase in pollen.

Figure S5.

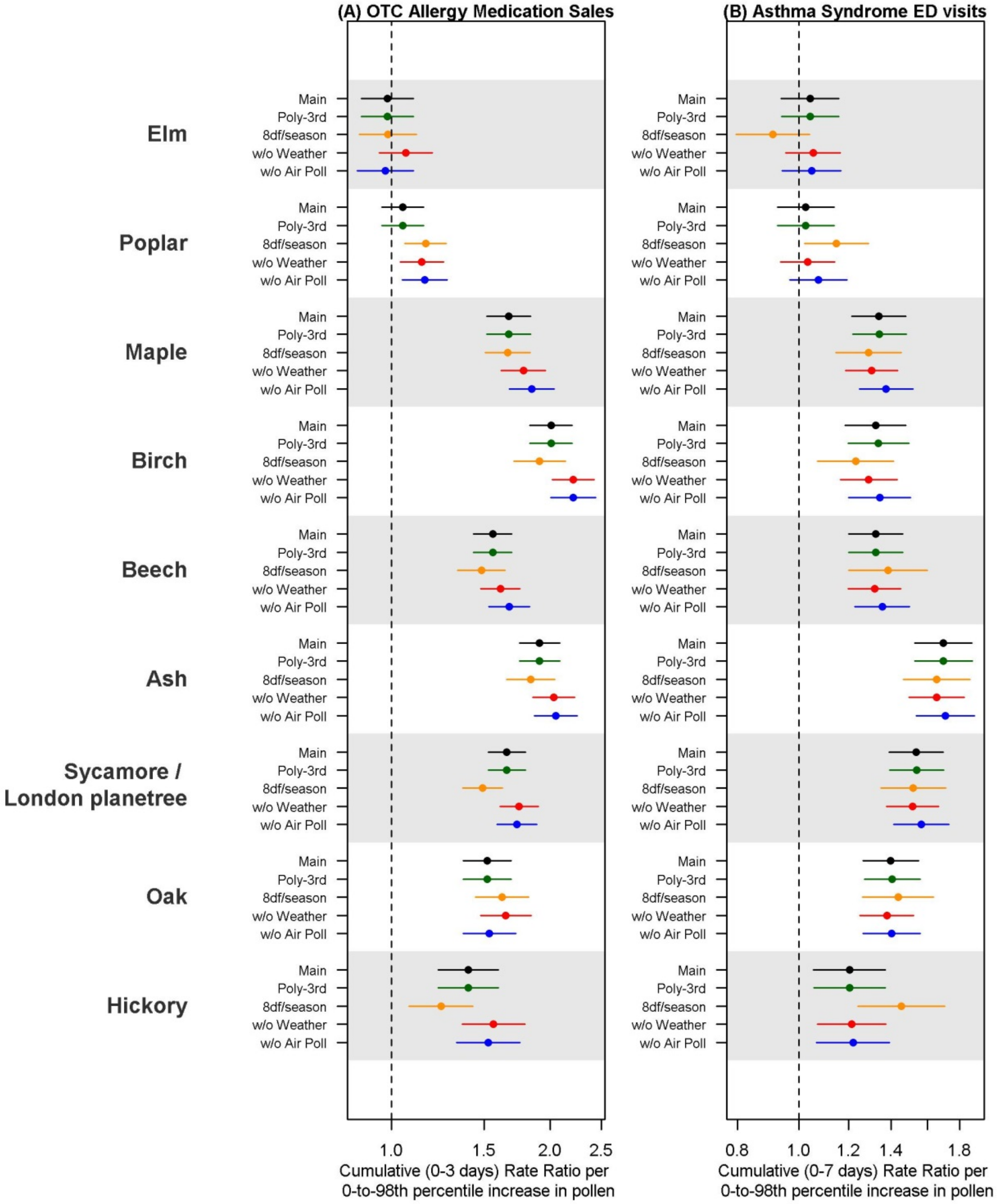

Sensitivity of estimated rate ratios to alternative model specifications for OTC allergy medication sales (left) and asthma syndrome ED visits (right). Models: (1): main analysis; (2) using a third-degree polynomial distributed lag (rather than the unconstrained lag in the main model); (3) using 8 degrees of freedom per season (rather than 6 df/season in the main model) for adjustment of temporal trends (4) without adjusting for weather terms; and (5) without adjusting for $PM_{2.5}$ and ozone. Rate ratios were computed per 0-to-98th percentile increase.
